# Investigation of cardiac fibroblasts using myocardial slices

**DOI:** 10.1093/cvr/cvx152

**Published:** 2017-08-18

**Authors:** Filippo Perbellini, Samuel A Watson, Martina Scigliano, Samha Alayoubi, Sebastian Tkach, Ifigeneia Bardi, Nicholas Quaife, Christopher Kane, Neil P Dufton, André Simon, Markus B Sikkel, Giuseppe Faggian, Anna M Randi, Julia Gorelik, Sian E Harding, Cesare M Terracciano

**Affiliations:** 1 Imperial Centre for Translational and Experimental Medicine, National Heart and Lung Institute, Imperial College London, Hammersmith Campus, Du Cane Road, London, UK; 2 Department of Cardiac Surgery, University of Verona, Verona, Italy; 3 Department of Cardiothoracic Transplantation and Mechanical Circulatory Support, Royal Brompton and Harefield NHS Foundation Trust, Harefield, UK

**Keywords:** Myocardial slices, Cardiac fibroblasts, Fibrosis, α-SMA, Mechanical load

## Abstract

**Aims:**

Cardiac fibroblasts (CFs) are considered the principal regulators of cardiac fibrosis. Factors that influence CF activity are difficult to determine. When isolated and cultured *in vitro*, CFs undergo rapid phenotypic changes including increased expression of α-SMA. Here we describe a new model to study CFs and their response to pharmacological and mechanical stimuli using *in vitro* cultured mouse, dog and human myocardial slices.

**Methods and results:**

Unloading of myocardial slices induced CF proliferation without α-SMA expression up to 7 days *in culture*. CFs migrating onto the culture plastic support or cultured on glass expressed αSMA within 3 days. The cells on the slice remained αSMA(−) despite transforming growth factor-β (20 ng/ml) or angiotensin II (200 µM) stimulation. When diastolic load was applied to myocardial slices using A-shaped stretchers, CF proliferation was significantly prevented at Days 3 and 7 (*P* < 0.001).

**Conclusions:**

Myocardial slices allow the study of CFs in a multicellular environment and may be used to effectively study mechanisms of cardiac fibrosis and potential targets.

## 1. Introduction

Progressive fibrosis is a hallmark of ageing in many organs.^1^ In cardiac tissue it results in stiffening of the myocardium and impaired relaxation with the development of diastolic dysfunction and heart failure in some patients.^2^ Cardiac fibroblasts (CFs), the principal regulators of the extracellular matrix (ECM), play a crucial role in the development of fibrosis, as well as contributing to the structural, mechanical, biochemical, and electrical properties of the myocardium.^3–5^ CFs are diffusely distributed throughout the myocardium and localized near large vessels.^6^ The proportion of CFs within the overall cellular composition of the heart is still debated due to the lack of specific markers and the variability between isolation protocols used.^7^^,^^8^ Vimentin (VIM), a Type III intermediate filament protein, is the most commonly used marker to identify CFs, despite it being non-specific and expressed by mesenchymal cells and some haematopoietic cells.^9^^,^^10^ Several stimuli, such as cytokines, cardiomyocyte death or changes in mechanical load, can activate CFs (activated CFs are also known as myofibroblasts).^11^ This activation is characterized by the expression of contractile proteins, especially alpha smooth muscle actin (αSMA),^12^ pathological remodelling of the myocardium and cytokine release. During pathological remodelling, CFs secrete excessive quantities of matrix proteins,^13^ including collagen Type I.^12^ Myofibroblasts also release a range of pro-inflammatory cytokines and these molecules can trigger CFs migration and proliferation, with an increase in CFs documented in the fibrotic heart.^14^ CF activation is driven by both autocrine and paracrine mediators from within the myocardium [angiotensin II (ANG II) and transforming growth factor β (TGF-β), circulating hormones (aldosterone) and changes in mechanical load].^11^ Exposure to fibrogenic growth factors (Platelet Derived Growth Factor and Fibroblast Growth Factor) and TGF-β is fundamental to CF activation as it stimulates αSMA expression and collagen synthesis, while suppressing ECM degradation.^15^ The origin of CFs in myocardial remodelling is debated. Previous studies have indicated that cells contributing to cardiac fibrosis are derived from the endothelium pericytes, bone marrow progenitor cells, monocytes and fibrocytes.^14^ However, a recent and comprehensive study by Kanisicak *et al*.^16^ using genetic lineage tracing, identifies in resident CFs the main source for activated myofibroblasts in the injured heart.

The study of CFs in intact hearts, whilst providing a physiological environment, does not allow direct observation of time-dependent changes and is too complex to determine cell origin, regulation, and fate; this is particularly true in human and large animals. Most studies on CFs have therefore been performed in cultures of isolated cells. However, this method results in rapid phenotypic changes,^17^ characterized by a significant increase in αSMA expression,^18^ changes in collagen and matrix protein deposition^18^ and CF electrophysiology.^19^ A variety of factors contribute to these culture-induced changes such as the stiffness of the material isolated CFs are cultured on,^20^ the use of different serum concentration in the culture media^21^ or varying oxygen levels.^22^ αSMA overexpression in studies with isolated cells is often used to identify myofibroblasts, as the phenotypic changes described are said to mimic CFs activation in pathology. However, this is highly debated, as many of these changes differ to those found in the heart *in vivo.*^18^ Given the highly plastic nature of CFs these differences are not surprising. The dramatic change in environment, from the cardiac tissue to cell culture of isolated cells, would be expected to produce different CF phenotypes.

Myocardial slices are 3D multicellular preparations that retain the cardiac cellular composition and ECM. Freshly prepared myocardial slices have been shown to have preserved structural, biochemical and electrophysiological properties.^23–25^ They have already been used as a promising experimental model to study mechano–electric interactions^24^ and *in vitro* drug safety screening.^26^ When cultured *in vitro*, they still display viability and their physiology is maintained in culture for 3 days after preparation suggesting that they are a good model to study acute and chronic effects of pharmacological, cell, gene, and other therapies.^27^^,^^28^ The ultra-thin dimension (200–400 µm) provides a 3D environment for the cells and also allows sufficient diffusion of oxygen and nutrients through the whole preparation, preventing ischaemic damage. Another advantage is the easy access to mid-myocardial tissue, which is challenging for larger preparations such as myocardial wedges or perfused whole hearts. The multicellularity, the 3D nature of the preparation and the presence of viable myocardium in culture, allow CFs to maintain physiological interactions with the other cardiac cells and the ECM.

Considering this, we hypothesized that the multicellular environment of myocardial slices could be used to prevent culture-induced changes in the CF phenotype *in vitro.* This would facilitate the investigation of the effect of mechanical and pharmacological stimuli on CF behaviour.

## 2. Methods

### 2.1 Heart collection

Human left ventricular transmural biopsies were obtained from the explanted heart of end-stage heart failure patients undergoing cardiac transplantation. Human samples were provided by the NIHR Cardiovascular Biomedical Research Unit at the Royal Brompton and Harefield NHS Foundation Trust and Imperial College London. The study performed conforms to the principles outlined in the Declaration of Helsinki and the investigation was approved by a UK institutional ethic committee (Ref: 09/H0504/104 + 5 CBRU Biobank). Informed consent was obtained from each patient involved in this study. Information regarding the human sample origin are summarized on *Figure 5G*. All animal experiments complied with UK Home Office standard regulations as designated by the EU Directive 2010/63/EU. The mice animal experiments were performed under the Home Office Project licence 70/7555. An 18-month-old healthy Beagle dog hearts were kindly donated by GlaxoSmithKline, after the animals were necessarily sacrificed at the end of pharmacology/toxicology studies. Only control animals were used in this study. The dogs were euthanized with an overdose of pentobarbital and the hearts removed immediately after confirmation of death. Both human and canine cardiac tissue blacks were quickly removed and immersed in 4 °C cardioplegia solution (Plegivex, Ivex Pharmaceuticals, UK; in mM: NaCl 147; KCl 16; MgCl2 16; CaCl2 1.2; Procaine hydrochloride), and transported to the laboratory (transport time ∼2 h). The transgenic mouse strain (Pdgfb-iCreER eGFP) used in these experiments were generated in Prof. Randi‘s laboratory accordingly to the protocol described by Claxton/Fruttinger.^29^ In these mice eGFP expression is dependent of the Pdgfb promoter. *In vivo* Pdgfb is predominantly expressed by endothelial cells and therefore were used this mouse model to clearly track and identify endothelial cells. The animals were kept under controlled conditions of temperature, light and humidity, with *ad libitum* access to water and chow. The mice were sedated with isofluorane, then sacrificed by cervical dislocation; death was confirmed by cessation of circulation. The heart was collected in 4 °C Tyrode Solution (140 mM NaCl, 6 mM KCl, 10 mM glucose, 10 mM HEPES, 1 mM MgCl_2_, 1 mM CaCl_2_; pH 7.4) containing 30 mM 2, 3-butanedione monoxime (BDM).

### 2.2 Myocardial slices preparation

An ∼1.5–2 cm^3^ transmural tissue block was cut from the left ventricular free wall of human and canine hearts using a razor blade (see Supplementary material online, *Figure S1A and B*). The trabeculae and papillary muscles were removed from the endocardium and the edges were then trimmed in order to obtain a final 1 cm^3^ tissue block (see Supplementary material online, *Figure S1C*). A thin layer of 4% agar was mounted onto the specimen holder and the tissue block was glued (Histoacryl, Braun, Germany) onto the agar with the epicardium (the flatter part of the block) facing down. The sample was placed on the cutting platform of a high precision vibrating microtome (7000 smz, Campden Instruments Ltd., UK), submerged in 4 °C oxygenated Tyrode Solution containing 30 mM BDM. We added BDM, an excitation–contraction uncoupler, to prevent the tissue from contracting while being sliced. The tissue was cut using a ceramic blade, longitudinal to the myofibril orientation, starting from the endocardium and progressively moving down to the epicardium. The vibrating microtome was programmed with settings optimized by Camelliti *et al.*^23^ The blade vibrated at 80 Hz with 2 mm amplitude and Z deflection <1 μm. 300 μm slices were obtained with the blade advancing at 0.04 mm/s. Once cut and prior to being cultured, slices were kept in 4 °C oxygenated Tyrode Solution with BDM under mesh holders (see Supplementary material online, *Figure S1E–G*).

### 2.3 Contractility

Slice contractility in response to field stimulation at 1 Hz was assessed using a force transducer (Harvard Apparatus, USA). 7 × 7 mm slices were prepared from the area with the most highly aligned myofibril orientation. To connect the slice to the force transducer, PTFE-coated silver rings holders were glued onto the edges of each slice, perpendicular to myofibril direction. The stimulation was conducted in a pre-warmed (37 °C) and pre-oxygenated perfusion chamber with Tyrode solution (with KCl 4.5 mM, and CaCl_2_ 1.8 mM). Maximum contractility was assessed at 1 Hz, with the stimulus width and voltage adjusted to ensure maximum contractility. The slice was progressively stretched in a step-wise manner, until maximum isometric contraction was obtained. Data were recorded by AxoScope software. Peak amplitude analyses were conducted using Clampfit software (Molecular Probes).

### 2.4 Tissue culture

#### 2.4.1 Liquid-air interface, transwell (unloaded)

Brandenburger *et al.*^30^ showed that long term slice viability could be achieved using an air-liquid interface. We utilized Transwell devices (Corning Incorporated, USA), semi-permeable inserts with 0.4 μm pores, within a 6-well dish. These provide a supporting platform for the slice, allowing full access to culture medium (CM) and oxygen (*Figure 7A*). Prior to being cultured, the slices were washed in phosphate buffered solution (PBS, Sigma) with 3% Penicillin/Streptomycin (Sigma). The myocardial slices were then placed on the Transwell membrane and any excess solution was removed from the slice surface and edges. 1 ml of sterile CM (Medium 199 (Sigma) + ITS [containing 1.0 mg/ml bovine insulin, 0.55 mg/ml human transferrin, and 0.5 μg/ml sodium selenite at the 100× concentration) (1: 1000) (Sigma) + Penicillin/Streptomycin (1: 100)] was added to each well. All cultures were kept in humidified air at 37 °C with 5% CO2 in an incubator. After 24 h the slices were washed 3 times with PBS and fresh CM was added; CM was changed every 3 days. Myocardial slices were kept for a maximum of 14 days in culture.

#### 2.4.2 Chemical stimulation

##### 2.4.2.1 TGF stimulation

culture media was supplemented with 10 or 20 ng/ml TGFβ, the slices were cultured on transwells using the TGFβ supplemented medium for 2 days. At the end of the stimulation the slices were washed, fixed with 4% paraformaldehyde (4%PFA) and stored in PBS at 4 °C.

##### 2.4.2.2 Angiotensin stimulation

culture media was supplemented with 200 µM ANG II. The myocardial slices were cultured on transwells and they were treated with ANG II medium for 4 days. The medium was replaced every 48 h. At the end of the stimulation the slices were washed, fixed with 4%PFA and stored in PBS at 4 °C. *VEGFα stimulation*: the myocardial slices were cultured on transwell for 3 days in CM supplemented with 20 ng/ml VEGFα. The medium was changed every day.

##### 2.4.2.3 Endothelial medium stimulation

the slices were cultured on transwell for 3 days. Endothelial Cell Growth Medium (Lonza, Swiss) was changed every day. At the end of the stimulation the slices were washed, fixed with 4%PFA and stored in PBS at 4 °C.

### 2.5 Fibroblasts isolation, fluorescent labelling, and *in vitro* culture

Enzymatic digestion was used to isolate CFs.^31^ Dog myocardial tissue was placed in ice-cold PBS and fat and connective tissue were removed. The tissue was cut into 1–2 mm^3^ pieces and transferred to a 15 ml Falcon tube. 5 ml enzymatic solution [Dulbecco‘s Modified Eagle Medium (DMEM) + penicillin/streptomycin (1: 100) + 0.1% Trypsin + 100 U/ml Collagenase V (Sigma)] was added. The tube was incubated at 37 °C in water bath for 15 min with occasional shaking. The isolated cells were then pooled, centrifuged, and re-suspended. Cells were cultured in DMEM supplemented with 10% FBS and penicillin/streptomycin (100 U/ml) and plated on bottom glass dishes (MatTek, USA). The dishes were kept in humidified air at 37 °C with 5% CO_2_ in an incubator for up to 10 days. After 1 h, the dishes were washed in sterile PBS to remove excess debris and dead cells. Medium was then replaced. This was repeated after 24 h. For the experiment described in *Figure 6I* and *J*, CFs were isolated (after enzymatic digestion, as previously described), the cells were labelled with QTracker 585 Cell Labelling Kit (Molecular Probes - Q25011MP) in line with the manufacturer‘s protocol. Labelled cells were then seeded on the surface of the myocardial slice and cultured for 1 week. Both labelled cells and the endogenous CFs proliferated on the slice surface; however, the presence of QTracker allowed the two cell types to be distinguished.

### 2.6 Live/dead assay

To assess the viability of cardiomyocytes on the slice surface, we performed a live/dead assay (LIVE/DEAD; Viability/Cytotoxicity Molecular Probes). To evaluate the number of living cells of the slice, myocardial slices were treated with trypsin for 5 min to remove the CFs proliferating on the surface of the slice. The assay was performed according to the manufacturer‘s instructions and imaged with the confocal microscope. A confocal microscope was used to acquire the images. Because of the density of the tissue, the laser could penetrate only the first two to three layers of cells and therefore the acquisition of data was limited to the surface of the preparation. To assess slice viability, we measured the percentage of area stained with the fluorescent Calcein-AM signal, the values were then expressed as normalized values on Day 0 slices.

### 2.7 Fixation and histological/immunohistochemical staining

Myocardial Slices were fixed in 4%PFA solution for 15 min and then washed in PBS. They were then permeabilized and blocked in 1% bovine serum albumin (Sigma) + 1% Triton X-100 in PBS for 2 h at room temperature on rocker. Slices were then incubated with primary antibodies, diluted in PBS, overnight at 4 °C, followed by a further three washes in PBS (30 min each). They were then incubated with secondary antibodies, diluted in PBS for 2 h at room temperature on rocker, with the samples covered in aluminium foil to prevent excessive light exposure. Slices were then washed another three times in PBS. Hoechst 33342 (1: 1000) in PBS was then added for 15 min at room temperature on rocker. Slices were washed a final time and then stored at 4 °C. All immunolabelled samples were then moved to bottom glass dishes and analysed using a Zeiss LSM-780 confocal microscope. Z-series of images were collected for each slice and combined for the final 2D reconstructed image. To investigate the centre of the slice, 15 µm sections of myocardial slices were prepared using a cryostat (Leica Biosystems, CM1850, UK). The cyosections were stained for PicroSirius red and four images per slice (*n* = 6) were acquired. Immunohistological staining was performed following the protocol described earlier. Images were processed with ImageJ (National Institutes of Health, USA). The percentage of slice occupied by CFs and the collagen content of cultured myocardial slices (see Supplementary material online, *Figure S2*) were quantified using ImageJ auto-threshold function and pixels quantification. The values were expressed as a percentage in respect to the analysed area.

**Table:** 

Primary antibody	Manufacturer	Dilution
Isolectin-B4 (Iso-B4)	Life Technologies – I21414 (Biotin conjugated)	1: 500
VIM	Abcam – ab92547 (Anti-Rabbit)	1: 2000
VIM	Thermo Scientific – PA1-16759 (Anti-chicken)	1: 3000
Caveolin3 (CAV3)	BD Bioscience (Anti-Mouse)	1: 500
αSMA	Dako – M0851 (Anti-Mouse)	1: 1000
Von Willebrand Factor	Abcam – ab6994 (Anti-Rabbit)	1: 500
Sarcomeric α-actinin	Sigma – A7811 (Anti-Mouse)	1: 1000
**Secondary antibody**	**Manufacturer**	**Dilution**
Alexa Fluor 555	ThermoFisher – S21381 (streptavidin)	1: 1000
CY3	Millipore – AP181C (Goat anti-Mouse)	1: 1500
Alexa Fluor 488	Life Technologies – A21206 (Donkey anti-Rabbit)	1: 1500
Alexa Fluor 546	Life Technologies – A10040 (Donkey anti-Rabbit)	1: 1500
Alexa Fluor 680	Life Technologies – A10043 (Donkey anti-Rabbit)	1: 1500
Alexa Fluor 680	Life Technologies – A21057 (Goat anti-Mouse)	1: 1500
Alexa Fluor 488	Life Technologies – A21202 (Goat anti-Mouse)	1: 1500

#### 2.7.1 Second harmonic generation (SHG) imaging to visualize and quantify myocardial slices collagen content

Collagen fibres were visualized using an adapted protocol described by Williams *et al.*^32^ An upright Leica SP5 confocal microscope, equipped with a Spectraphysics Mai Tai 690-1020 DeepSee multiphoton laser were used to performed SHG. The multiphoton was tuned to 850 nm for Collagen I (SHG) detection at 410–430 nm. Samples were mounted in dishes and Z-series of images were collected with dry and water dipping objectives. Images were acquired with a 10× and 25× magnification and processed with ImageJ (National Institutes of Health, USA).

#### 2.7.2 Sarcomere length quantification

myocardial slices were stained for sarcomeric α-actinin, and imaged with confocal microscopy. The images were processed with ImageJ software (NIH). A line was drawn perpendicularly to several consecutive sarcomeres, the sarcomeric α-actinin resulted in peaks on the fluorescence signal which corresponded to the consecutive sarcomeres. The length of the line was then divided by the number of peaks. Three images per slice were acquired and the sarcomere length of at least five cells per image was measured.

### 2.8 Statistical analysis

Statistical comparison between groups was performed using a student-unpaired *t*-test or one way analysis of variance followed by Tukey‘s test using Prism5 software (GraphPad, San Diego, USA). When multiple slices from multiple animals were considered, statistical analysis was performed using hierarchical statistical techniques (random intercept mixed model) as per Sikkel *et al*.^33^*P* values *< 0.05, **<0.01, and ***<0.001 were considered statistically significant.

## 3. Results

### 3.1 Cellular composition of intact myocardial slices vs. after enzymatic digestion

**Figure 1:**
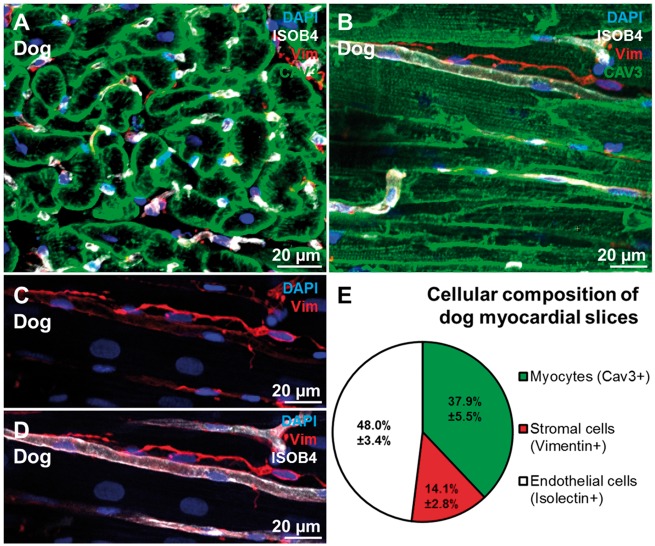
Cellular composition of dog myocardial slices. (*A,B*) transverse and longitudinal section of a myocardial slice; cardiomyocytes, endothelial cells and fibroblasts were stained for CAV3 (green), ISOB4 (white), and VIM (red). (*C,D*) Higher magnification of CFs (red) and their location in proximity to capillaries (white). (*E*) The cardiac cellular composition is made up by 48% endothelial cells, 14.2% stromal cells, 37.9% myocytes. 44 images generated from 10 myocardial slices were processed and a total of 3259 cells were counted.

**Figure 2:**
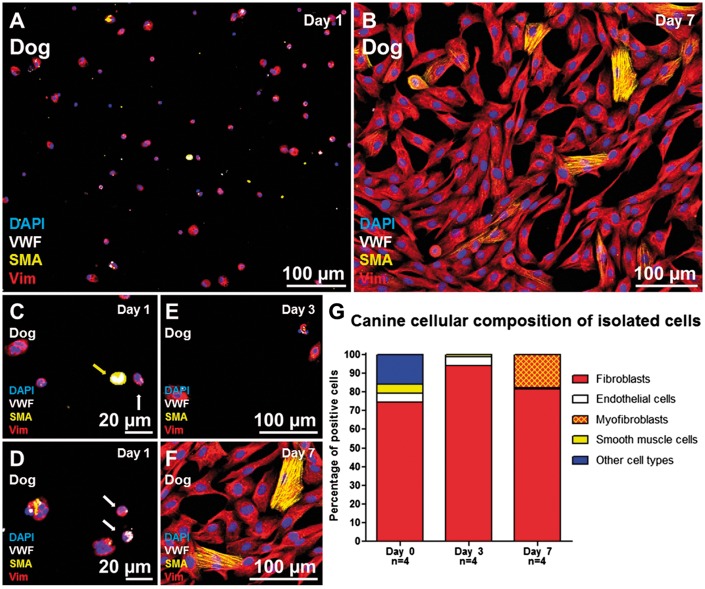
Cellular composition of isolated cells after enzymatic digestion of myocardial tissue. (*A,B*) Isolated cells at Days 1 and 7 stained for VIM (red), αSMA (yellow), and VWF (white). (*C–F*) higher magnification of isolated cells, the yellow arrow indicates a smooth muscle cell (SMA+), the white arrows indicate endothelial cells (VWF+) (*G*) after enzymatic digestion 74.5% of the isolated cells were CFs (VIM+/αSMA−/VWF−), 5% were smooth muscle cells (VIM−/αSMA+/VWF−) and 4.5% were endothelial cells (VIM−/αSMA−/VWF+). The remaining 16% of the cells were negative for all the antibodies (VIM−/αSMA−/VWF−) and included cardiomyocytes and other cell types. After 10 days in culture almost exclusively VIM+ cells survive and the cells start to express αSMA (yellow). Two bottom glass dishes were prepared for each time point of each isolation (four isolations) and averaged before statistical analysis.

A 300 µm thick freshly prepared dog myocardial slices (see Supplementary material online, *Figure S1*) were fixed in 4%PFA. The samples were not subjected to freezing or sectioning in order to minimize tissue damage and to preserve the cardiac structure and cellular composition. The main three cell populations in the heart are cardiomyocytes, endothelial cells and stromal cells (the majority of which are CFs). The canine cardiac cellular composition was investigated using the three antibodies CAV3, VIM, and Iso-B4 (*Figure 1A–D*). The CAV3 antibody provided a clear membrane staining which was used to distinguish and count the cardiomyocytes; endothelial cells were identified with the Iso-B4 a lectin-based histochemical strategy that clearly labelled endothelial cells. VIM was used to identify stromal cells. Although VIM is a non-specific CF marker, it is commonly used to identify CFs in the literature.^9^ In this study, VIM+ cells will be considered as CFs and the limitations of this will be discussed later. The quantification of myocardial slice cell composition showed that endothelial cells are the predominant cell population in the heart (48%) followed by cardiomyocytes (37.9%) and stromal cells (14.1%) (*Figure 1E*). The cardiac stromal cells were isolated from 1 to 2 g of dog left ventricular myocardium with a well-established method of consecutive enzymatic digestions.^34^ After collagenase digestion the cells were seeded on bottom glass dishes and fixed after 12 h or cultured *in vitro* until Days 3 or 7 (*Figure 2A–F*). At the first time point (12 h) 74.5% of the isolated cells were CFs (VIM+/αSMA−/VWF−), 5% were smooth muscle cells (VIM−/αSMA+/VWF−), 4.5% were endothelial cells (VIM−/αSMA−/VWF+), and 16% were other cell types and negative for all the antibodies (VIM−/αSMA−/VWF−). After few days in vitro the VIM+ cells started to grow in size, the numbers of endothelial cells and smooth muscle cells decreased with time in culture and some of the VIM+ cells acquired a myofibroblast phenotype characterized by αSMA-enriched stress fibres expression (*Figure 2G*).

### 3.2 Temporal relationship between CF proliferation and culture-induced changes in myocardial slices

**Figure 3:**
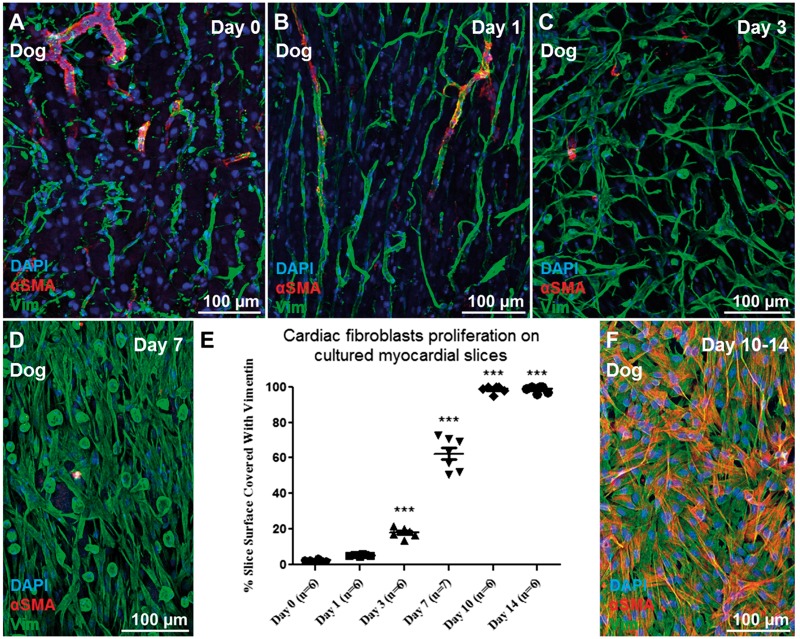
CFs proliferation on myocardial slices. Representative images of (*A–D*) VIM+ cell (green) proliferation on the myocardial slice with time. (*E*) We observed a significant increase in the percentage of the slice surface covered by VIM+ cells at Day 3, with a further significant increase seen at Days 7 and 10. (*F*) By Day 10 the cells completely covered the surface of the slices and started to express αSMA (red). Three images per slice were acquired and the data obtained were averaged before statistical analysis. Six slices were used for this experiment.

Myocardial slices were prepared from canine and human left ventricular myocardium and cultured *in vitro* on a liquid-air interface. Using this method the slices were cultured under unloading conditions, which have been shown to stimulate CF proliferation *in vivo*.^35^ VIM+ cells proliferated on myocardial slice surface (*Figure 3A–D*) and a significant increase in the percentage of the slice surface was covered by VIM+ cells at Day 3 (*P* < 0.001) and a further significant increase at Day 7 (*P* < 0.001) was observed (*Figure 3E*). By Day 10 the whole slice surface was covered with VIM+ cells (*Figure 3E*). To investigate whether the CFs were migrating and proliferating exclusively onto the slice surface or also within the centre of the preparation, 15 µm sections were prepared using a cryostat and the sections were analysed with immunohistochemistry. Our data show that CFs proliferated not only on the surface of the myocardial slice, but also within the centre of it (see Supplementary material online, *Figure S2A–E*). Using histological sections and SHG imaging the collagen content of freshly prepared and Day 7 myocardial slices was quantified (see Supplementary material online, *Figure S2F–K*). No differences were noticed between the two groups suggesting that the *in vitro* culture of myocardial slices induce CF proliferation without affecting the ECM collagen content.

**Figure 4:**
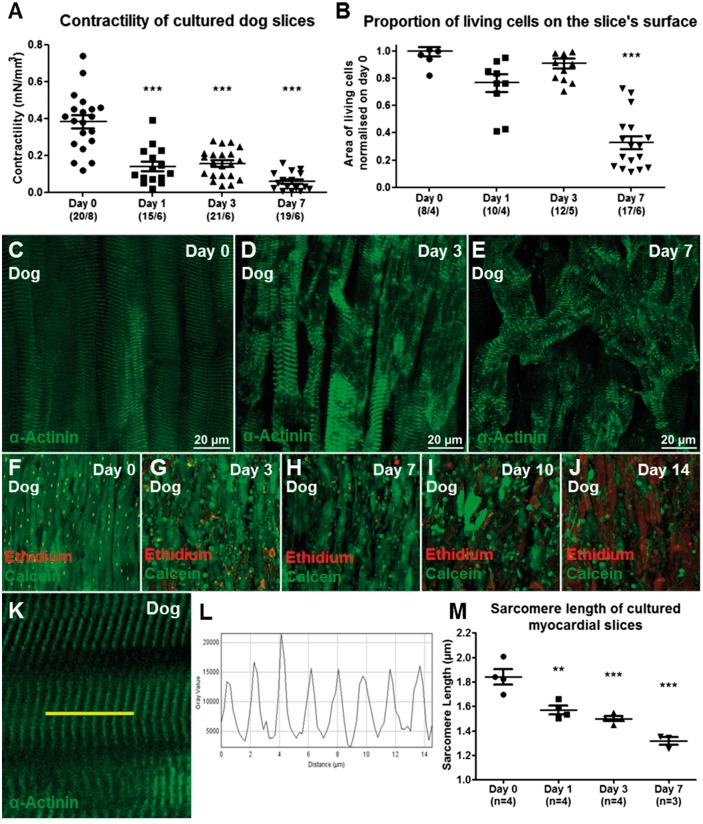
Characterization of myocardial slices. The slices responded to prolonged in vitro culture under unloading conditions (*A*) with significantly decreased contractility (36%) for the first 3 days and progressively deteriorated to 15% by Day 7. Number of slices/number of experiments. (*B, F–J*) The viability of the slice was preserved until Day 7, when a significant decrease in the area of living cells was measured (three images per slice were acquired and the data obtained were averaged before statistical analysis. Number of slices/number of experiments; *P* < 0.001). Immunofluorescence staining for sarcomeric α-actinin (Green) showed myocytes size changes and sarcomeric reorganization occurs with time, suggesting cardiomyocytes dedifferentiation (*C–E*). The sarcomere length of myocardial slices showed a significant decrease within 24 h in culture (9 *K–M*). Three images per slice were acquired and the sarcomere length of at least five cells per image was measured, the data were averaged before statistical analysis.

As previously reported for other species, such as rat and human,^5^^,^^36^ isolated CFs start to express the myofibroblasts marker αSMA+. On the contrary, in this model αSMA expression was absent in VIM+ cells proliferating on myocardial slice surface for the first 7 days. Only at Day 10 VIM+ cells had filamentous αSMA+ stress fibres, suggesting their transformation into a myofibroblast phenotype (*Figure 3F*). Live/dead assay was performed to assess slice viability. The viability of the slice was preserved until Day 7, when a significant decrease in the area of living cells was measured (*P* < 0.001) (*Figure 4B, F–J*). Consistent with this, sarcomeric α-actinin staining revealed tissue remodelling with reorganization of the sarcomere structure, probably due to cell dedifferentiation (*Figure 4C–E*). Freshly prepared dog myocardial slices showed a maximum contractility of ∼0.4 mN/mm^3^. As expected and previously reported^30^ the slices responded to prolonged *in vitro* culture under unloading conditions with significantly decreased contractility (36%) for the first 3 days and progressively deteriorated to 15% by Day 7 (*Figure 4A*).

**Figure 5:**
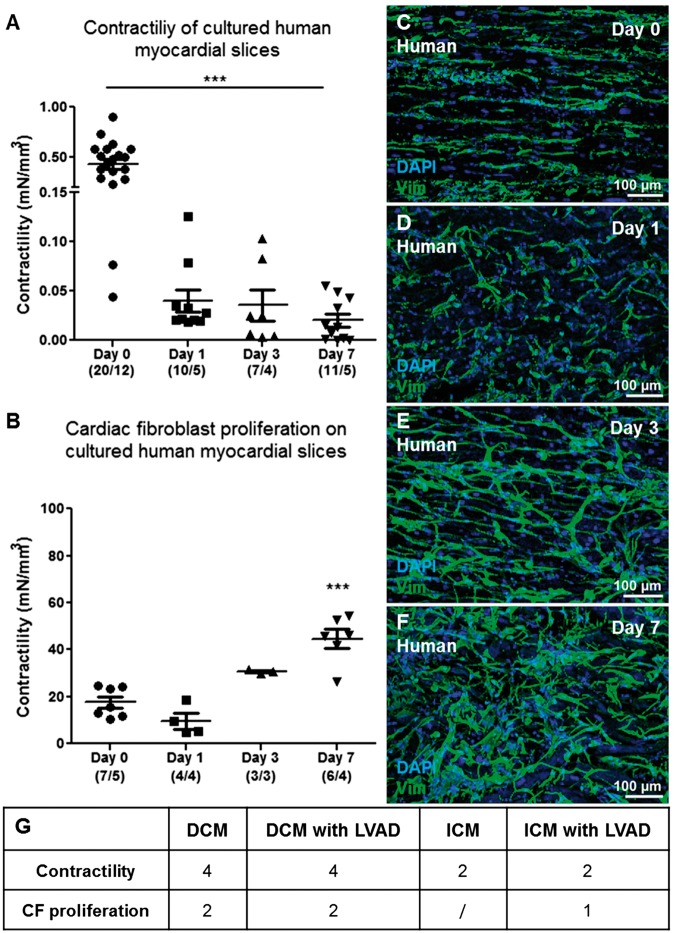
CFs proliferation and contractile capacity of culture human myocardial slices. Human myocardial slices cultured in vitro progressively lose their contractility (*A*). Number of slices/number of experiments. As observed in canine myocardial slices, we noticed a significant increase in the percentage of the slice surface covered by VIM+ cells (*B*). Representative images of VIM+ cells proliferating on the human slice surface at Days 0, 1, 3, and 7 (Green, *C–F*). Three images were acquired from each slice and the data obtained were averaged before statistical analysis. The slices were prepared from at least three human samples (K). The Table summarizes the groups of end-stage heart patient biopsies used in the study. (G) DCM, dilated cardiomyopathy; DCM with LVAD, dilated cardiomyopathy with left ventricular assist device; ICM, ischaemic cardiomyopathy; ICM with LVAD: ischaemic cardiomyopathy with left ventricular assist device.

The sarcomere length of freshly prepared and cultured slices was assessed and revealed a significant decrease within 24 h (*Figure 4K–M*). Similar observations were made on human myocardial slices prepared from human end-stage heart failure biopsies. 12 human samples were used for this study. *Figure 5K* summarize the groups of end-stage heart patient biopsies used in the study. Freshly prepared human slices showed a maximum contractility comparable to dog slices (∼0.4 mN/mm^3^) but a more pronounced decrease in contractility after 24 h of *in vitro* culture (*Figure 5A*). As described for dog samples, VIM+ cells proliferated on the myocardial slice surface (*Figure 5B*) and did not express the myofibroblast marker αSMA at Day 7 (*Figure 5C–F*).

For both human and dog slices, a higher density of proliferating VIM+ cells was noted around larger blood vessels and the cells appeared to migrate away from the vessels (see Supplementary material online, *Figure S3A, C* and *D*). VIM+ cells also proliferated out of the interstitial space. To determine if the VIM+ cells that proliferated from perivascular sources were derived from the vascular endothelium after endothelial-mesenchymal transition (EndMT) we used a Pdgfb-iCreER transgenic mouse strain^29^^,^^37^ (see Supplementary material online, *Figure S3B*). In this mouse model eGFP expression is Pdgfb dependent and therefore restricted to endothelial cells, allowing cell-specific lineage tracing. Day 7 mouse slices were analysed with immunofluorescence staining and, as observed for dog samples, proliferating fibroblasts were found on the surface of the slice (see Supplementary material online, *Figure S3E, G–I* and *S4*). These cells were GFP negative suggesting that they do not derive from the vascular endothelium via EndMT.

### 3.3 Isolated CFs seeded on myocardial slices proliferated in culture and did not express αSMA

**Figure 6:**
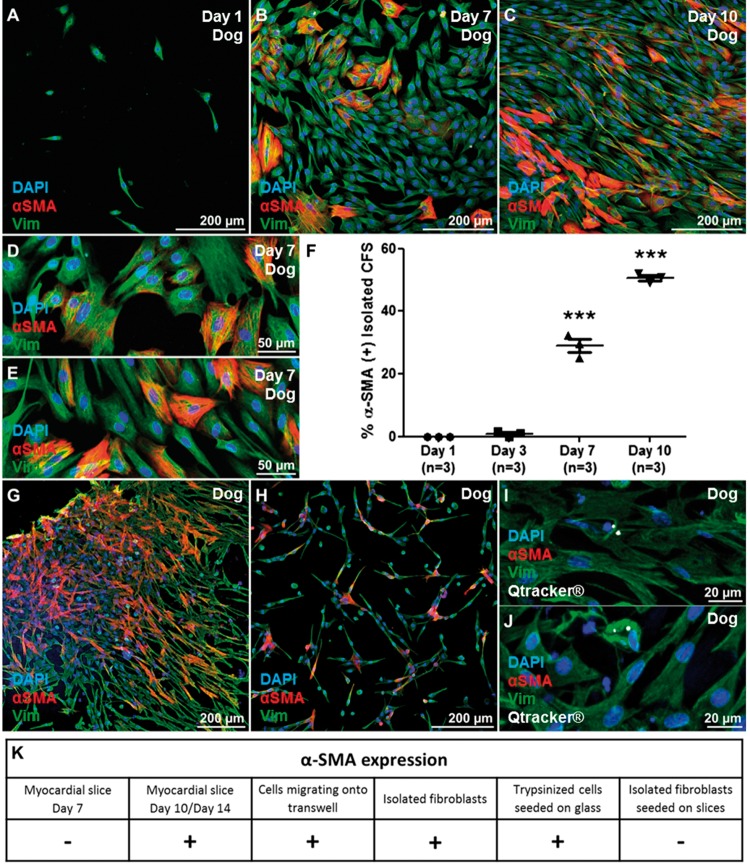
Isolated CFs *in vitro*. Freshly isolated dog CFs (VIM+/αSMA−), were seeded on bottom glass dishes coated with fibronectin. (*A–E*) Within 3 days, CFs started to express αSMA. By Day 7 30% of the cells were αSMA+ and the number increased to 50% by Day 10 (*F*). The experiment was repeated three times and three images were acquired from each dish and the data obtained were averaged before statistical analysis. VIM+ cells that proliferated out of the myocardial slices and onto the Transwells membrane expressed αSMA at Day 7. (*G*). VIM+ cells, trypsinized from Day 7 slice surface and cultured on glass, expressed αSMA after 24 h (*H*)*.* The cells were isolated after collagenase digestion, labelled with Qtracker 585 (white) and seeded onto dog slices (*I, J*). Labelled CFs were then cultured for 7 days and observed to be αSMA−. (*K*) CF αSMA expression was assessed in different conditions. CF proliferating on the slice surface were αSMA(−) after 7 days of *in vitro* culture. CFs migrating onto the culture plastic support or isolated with enzymatic digestion and cultured on glass were αSMA(+). CFs proliferating on myocardial slices for 7 days [αSMA(−)] were trypsinized and seeded onto glass dishes and on myocardial slices. These cells were respectively αSMA(+) and αSMA(−) suggesting a crucial role in the slice as a substrate to preserve a more physiological phenotype. Each experiment was repeated at least three times.

Canine CFs were isolated after collagenase digestion and seeded onto glass bottom dishes coated with fibronectin. The cells adhered to the dish and proliferated *in vitro*. As previously reported for other species, such as rat and human,^5^^,^^36^ within few days post isolation CFs started to express the myofibroblasts marker αSMA+ (respectively 1, 29, and 50% at Days 3, 7, and 10) (*Figure 6A–F*). Considering CFs that proliferated on myocardial slices lacked αSMA, we investigated whether the myocardial slice had properties that were preventing αSMA expression in VIM+ cells. We studied VIM+ cells that migrated from the slice and onto the Transwells membrane. At Day 7, VIM+/αSMA+ cells were observed proliferating on the Transwells membrane (*Figure 6G*). Additionally, VIM+/αSMA− cells were trypsinized from the myocardial slice surface and cultured on glass. After 24 h the cells began to express αSMA *in vitro* (*Figure 6H*). In order to determine if the lack of αSMA expression in VIM+ cells was due to specific features of these proliferating cells or the properties of the myocardial slice, we seeded isolated CFs labelled with fluorescent nanocrystals (Qtracker585) on the surface of dog myocardial slices. At days 1, 3 and 7, the isolated CFs remained VIM+/αSMA- (*Figure 6I*).

### 3.4 Pharmacological treatments and mechanical stimulation of myocardial slices

To determine whether αSMA expression could be induced in VIM+ cells proliferating on the myocardial slice surface, we stimulated slices with TGF-β and ANG II, both potent fibroblast activators.^15^ Slices were stimulated with 10 and 20 ng/ml of TGF-β for 2 days. These concentrations are known to induce αSMA expression in isolated cultures.^38^ The slices were also treated with 200 µM ANG II for 4 days. The drugs did not induce CF proliferation and interestingly the cells did not express αSMA after the pharmacological stimulations (*Figure 8A–C*). At the end of the pharmacological treatment (4 days), whilst the CFs on the slice remained αSMA−, the cells that migrated onto the Transwells expressed αSMA (*Figure 8B*).

Mechanical load is a key regulator of CF structure and function.^12^ CF proliferation and ECM deposition are two key events that occur during mechanical overload^39^ and unloading of cardiac tissue *in vivo.*^40^ A range of uniaxial stretch (between 5 and 20%) have been shown to induce changes in CFs proliferation, gene expression and ECM deposition *in vitro.*^41^^,^^42^ When cultured on Transwell membranes, myocardial slices are unloaded. The lack of mechanical load may have been one of the factors that activated CFs and induced them to proliferate on myocardial slices. To test this we developed 3D printed A-shaped stretchers to apply a physiological 10% static mechanical load to myocardial slices for an extended period of time (up to 7 days).

**Figure 7:**
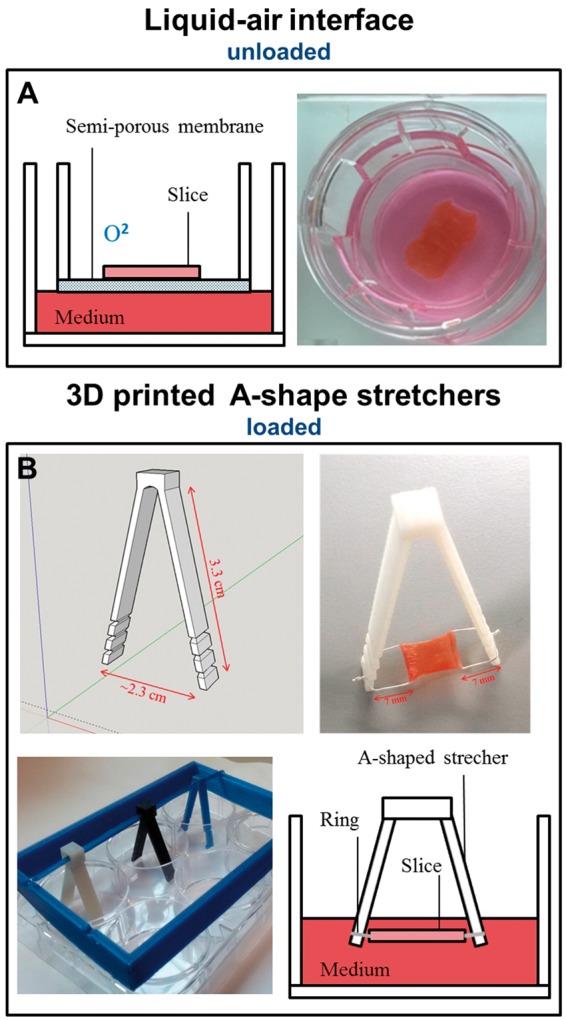
Graphical and photographic representation of myocardial slices culture method. Transwells membrane (*A*) were used for the liquid–air interface culture method. With this culture condition the slices were mechanically unloaded. (*B*) 3D printed A-shape stretchers. These devices were used to apply mechanical load to myocardial slices. PTFE-coated silver rings holders were attached to the edges of each slice, perpendicular to myofibril direction. The lateral notches allow a progressive stretch of the slice.

**Figure 8:**
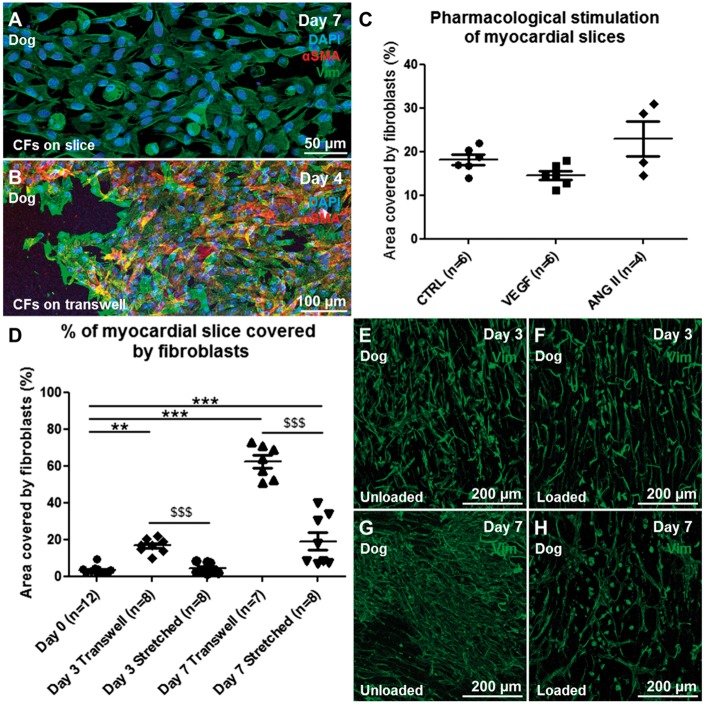
Pharmacological and mechanical stimulation of myocaridial slices. (*A*) The pharmacological stimulation of myocardial slices with pro-fibrotic drugs at concentrations that induce myofibroblast phenotype in isolated CFs did not induce αSMA expression in the proliferating CFs. (*B*) VIM+ cells that proliferate out of the myocardial slice (VIM+ cells, green) and onto the Transwells membrane express αSMA (red). (*D*) Mechanical stretch of myocardial slices induced a significant reduction in fibroblast proliferation at both Days 3 and 7. Representative images of CFs stained for VIM (green) proliferating on the surface of myocardial slices at Days 3 (*E* unloaded, *F* loaded) and 7 (*G* unloaded, *H* loaded). Each experiment was repeated at least three times, to quantify CF proliferation three images per slice were acquired and the data obtained were averaged before statistical analysis.

A scalpel was used to trim freshly prepared myocardial slices to a dimension of 7 × 7 mm^2^; PTFE-insulated silver (Ag) wire (Goodfellow, Chambridge, UK) rings were glued to the edges of the slice, oriented perpendicularly to the cardiac muscle fibres. The silver rings allowed the slices to be hung on A-shaped stretchers (*Figure 7B*). SketchUp Make software (Trimble navigation ltd) was used to design the A-shaped stretchers which were 3D printed using the FDA approved polymers Taulman T-glase PETT Blue (3D Prima, Sweden). The stretchers have lateral notches to allow a progressive stretch of the slice to a final 10% increase in the slice length. The slices were cultured for up to 7 days in CM. The application of mechanical load significantly reduced CF proliferation at Days 3 and 7 (*P* < 0.001) (*Figure 8D–H*). The application of mechanical load to myocardial slices also had effects of the whole myocardial slice. These included improved sarcomeric structure and tissue organization/architecture, as shown with α-actinin staining of myocardial slices in see Supplementary material online, *Figure S2L* and *M*. After 7 days in culture αSMA is not expressed in either loaded or unloaded slices. The collagen content of myocardial slices cultured in unloaded or loaded conditions for 3 days was quantified using SHG imaging (see Supplementary material online, *Figure S2N–P*). No differences in the collagen content were observed between the two groups suggesting that the mechanical load doesn’t induce ECM deposition.

## 4. Discussion

In this study, we set out to determine if the multicellular environment of myocardial slices could prevent culture-induced changes in CF phenotype *in vitro*. Our results demonstrate that: (i) human and canine myocardial slices can be kept in culture and can be used to study endogenous CFs in a more physiological approach than culture of isolated cells; (ii) CFs proliferating on myocardial slices do not express αSMA after 7 days in culture, whereas CFs cultured on glass do; (iii) αSMA expression in CFs on myocardial slices could not be stimulated by TGF and Angiotensin at doses that would induce this expression in isolated CFs; (iv) mechanical load applied to slices can be used to modulate CFs proliferation. We have also shown that myocardial slices can be used to investigate the cardiac cellular composition.

### 4.1 CFs studied on cultured myocardial slices

As previously described using a liquid-air interface culture system, myocardial slices can be cultured *in vitro.*^27^^,^^30^

In this study, we observed activation of CFs and their proliferation on the slice surface and within the centre of the slice. We showed a significant increase in the percentage of the slice surface covered by VIM+ cells at Day 3, with a further significant increase seen at Day 7 in both human and canine myocardial slices. In this study, we used VIM as a marker for CFs. As VIM is not specific for CFs only and we wanted to make sure that the proliferating cells were mostly CFs, we tested two VIM antibodies from two different companies (Sigma and Thermo Scientific) and the florescence signal overlapped. We also combined VIM with the more specific CFs marker S100A4 (Fibroblast-specific protein 1) antibody. Most of the cells were double positive confirming the CF origin of the proliferating cells (see Supplementary material online, *Figure S5*).

Surprisingly CFs cultured on myocardial slices for up to 7 days did not express the myofibroblast marker αSMA. As previously described in Ref. 4 isolated CFs cultured on glass started to express αSMA within few days in culture. When VIM+ cells lose contact with the myocardial slice, either by migration onto the Transwells membrane or by trypsinization from the slice surface, rapid αSMA expression occurs. When this is coupled with our observation that isolated fibroblasts seeded on myocardial slices remain αSMA(−) at Day 7, it appears myocardial slices have properties that prevent αSMA expression in VIM+ cells in culture. By Days 10–14 the CFs proliferating on myocardial slices are αSMA+. There are several possible explanations for this, including the high cellular density which CFs encounter during the *in vitro* proliferation, the increase in the number of dead cells on the slice and possible changes to the stiffness of the slice. All these factors are known to affect αSMA expression *in vitro*^20^^,^^39^; however, further studies are required to better investigate these processes. Next we investigated the viability and functionality of *in vitro* cultured myocardia slices and we showed that although human and canine myocardial slices display a good viability and contractility during the first 3 days in culture, by Day 7 there is a significant decline in term of function and number of viable cells. As expected, during *in vitro* culture both cardiomyocytes and CFs respond to the unloaded condition and *in vitro* culture environment. Cardiomyocyte contractile dysfunction is a physiological adaptation of resting cardiomyocytes to the absence of mechanical load, which affects cardiomyocytes both *in vitro* and *in vivo.*^43^ The significant decrease in sarcomere length of slices cultured for 24 h confirmed an involvement of the contractile apparatus; however, further studies are necessary to better understand and characterize the mechanisms. In this study, human data were collected from end-stage heart patient biopsies. Four samples originated from Dilated Cardiomyopathy (DCM), four from DCM with Left Ventricular Assist Device (LVAD); two from Ischaemic Cardiomyopathy; and two from ICM with LVAD. The various groups were not divided because of the low N number. Furthermore we did not observe obvious differences between the groups in terms of contractility or CF proliferation. The main difference observed in healthy control dog slices in comparison to myocardial slices prepared from end-stage heart failure biopsies was the enhanced reduction in contractility after 24 h in culture. These results may suggest that the end-stage pathological condition of the tissue, which involve deprived metabolic state, structural degradation and enhanced fibrosis, could result in a reduced adaptive response to the *in vitro* culture environment.

Recent findings from Kang *et al.*^27^ showed that *in vitro* culture degraded the electrical properties of human slices prepared from failing hearts; however, the same observation was not found in healthy human myocardial slices.

We noticed that a high densities of VIM+ cells proliferated around larger blood vessels. VIM+ cells that proliferate from perivascular sources can be derived from a number of cell sources.^44^ Recent studies on the vascular endothelium show that it is involved in the fibrosis, with 27–35% of all fibroblasts derived via EndMT.^44^ To determine if proliferating cells on myocardial slices are derived from the vascular endothelium, we stained for VWF and our results showed VIM+ cells, proliferating from perivascular sources, do not express VWF (data not shown). We then used hearts from Pdgfb-iCreER transgenic mice.^29^ In this mice, eGFP expression is restricted to endothelial cells allowing cell-specific lineage tracing. Day 7 mouse slices were analysed with immunofluorescence staining and proliferating CFs were found on the surface of the slice; these cells were GFP(−) suggesting that, as suggested by Molkentin *et al*.,^16^ they might derive from the endogenous fibroblasts population rather than from EndMT process. However, these results do not completely rule out the possibility that these cells are derived from the endothelium, as rapid loss of VWF or Pdgfb can occur during the transition to a fibroblast-like phenotype. Fate-mapping studies have also identified the epicardium and circulating cells (bone marrow derived cells,^44^ fibrocytes,^45^ and monocytes^46^) as sources of CFs. Considering that the myocardial slices used in the study were not produced from the epicardium and cessation of circulation has occurred, these seem unlikely sources. It has also been suggested that pericytes may contribute to cardiac fibrosis, although this is yet to be investigated.^47^

We also tried to stimulate proliferating VIM+ cells to express αSMA with ANG II and TGF-β, at doses well known to have such effects *in vitro.*^15^ CFs on slices did not express αSMA. Although surprising, there are a number of plausible explanations. First, the concentration of TGF-β used may not have been high enough as the concentrations referenced are commonly used to induce αSMA expression in isolated CFs in a cell monolayer^38^ further suggesting that culture of isolated cells in monolayers may not be adequate for pharmacological studies of myocardial fibrosis. In comparison, myocardial slices contain many thousands fibroblasts (∼112 000), as well as cardiomyocytes and other cell types. Another possible reason could be that protective paracrine factors, released by the slice, are not overcome at these concentrations. In order to investigate this further, we plan to stimulate the slice at higher concentrations and for longer periods.

A crucial observation from our study is that, under normal physiological circumstances, resident CFs do not express the contractile protein αSMA^48^ and it is only following pathological insult or mechanical unload that CFs proliferation, activation and αSMA expression occur. Because on slices CFs proliferation occurs in a robust fashion, there must be a difference between the stimulus for VIM+ cells to migrate and proliferate, and then to subsequently express αSMA. A recent paper from Tillmanns *et al*.^49^ shows the important role of the Fibroblast activation protein α in inducing in CFs a migratory proto-myofibroblast phenotype which have F-actin stress fibre network without αSMA incorporation. Another possible explanation for this phenomenon could be a change in paracrine communication or electrotonic interactions^50^ between or among CFs or between CFs and other cell types such as myocytes. We cannot exclude the possibility that the myocytes become depolarized in a progressive time-dependent fashion and this could affect CF proliferation and or αSMA expression. Further studies are required to better investigate this aspect.

*In vivo* the myocardium is constantly mechanically loaded, but in culture, using the liquid–air interface method, the slices become completely unloaded. The increasing use of LVADs^51^ has permitted the study of mechanical unloading on cardiac fibrosis *in vivo.* A definitive study by Drakos *et al.*,^35^ utilizing digital histopathology, has revealed increased interstitial and total fibrosis in response to unloading. Our results have clearly shown that mechanical unloading is a key regulator in CFs proliferation and the application of load can reverse this process. These findings support the use of myocardial slices as a tuneable platform to further investigate the mechanism and signalling involved in cardiac fibrosis. Taken into account that myocardial slices represent an intermediate level biological model system and they have a pseudo 2D to 3D configuration, it has been suggested^50^ that they could be used to study electrotonic cell interaction. The possibility of manipulating mechanical load, and therefore mimicking conditions of physiological load or pathologic unloading and overload, will further enhance the relevance of these findings.

### 4.2 Slice cellular composition and enzymatic cell isolation

Despite an extensive number of studies, there is not a definitive consensus regarding the contribution of each major cardiac cell type on the total cellular composition of the heart.^52^ Here, using confocal microscopy, we demonstrated that myocardial slices are a viable and minimally damaged cardiac preparation. Confirming the findings from some recent studies in other species,^3^^,^^53^ we demonstrated that also in canine myocardium endothelial cells are the most abundant cell population in the heart and the stromal composition is much smaller than that frequently reported in the literature.^8^^,^^36^^,^^47^ Although the staining and imaging was performed on the entire myocardial slice, the antibodies were able to penetrate only one to three cell layers into the tissue (roughly 20–30 µm); therefore, the acquisitions were limited to the surface of the samples. Multiple consecutive images were acquired in a Z-stack and then compressed into a 2D image, the cell count was performed analysing every single colour and then combining the colours to better distinguish the different cell types. This process does not require tissue digestion which can affect cell types differently, but does not have the same high throughput as flow cytometry. To compensate, 44 images generated from 10 myocardial slices were processed and a total of 3259 cells were counted. Another limitation is that only three antibodies were used in this study. This approach does not differentiate sub-populations but is adequate to quantify the three main cardiac cell types. Considering the viability of the cells on the surface of the preparation and the transmural origin of the slices, this preparation is a reliable representation of the adult myocardium. This approach eliminates the issues of selecting the stronger cells which is the main limiting factor of enzymatic cell isolation. After collagenase digestion our data were similar to what is reported in the literature^8^^,^^39^ in showing that ∼70% of the cells are VIM+. This number increased with cell culture, suggesting that the *in vitro* culture may be selecting and promoting a specific cell type expansion.

## 5. Conclusions

Living myocardial slices constitute a multicellular environment of intermediate complexity which can be cultured and manipulated *in vitro* to investigate the biology of CFs in different species. In this study we have shown that, using human and canine myocardial slices, CFs can be kept in culture for several days without observing culture-induced α-SMA expression and their proliferation can be modulated with the application of mechanical load, resembling physiological behaviour *in vivo*. These observations support the use of myocardial slices to better understand CFs function and regulation and to study mechanisms of cardiac fibrosis and electrotonic interactions.

## Supplementary Material

cvx152_Supplementary_Data

## Notes

Time for primary review: 43 days
