# Supplementary material for: Investigation of cardiac fibroblasts using myocardial slices
Source: Cardiovasc Res. 2017 Aug 18;114(1):77–89. doi: 10.1093/cvr/cvx152 (PMC5852538; doi:10.1093/cvr/cvx152)
Supplement: cvx152_Supplementary_Data [file cvx152_supplementary_data.docx]

**Supplementary data**

**SUPP FIG 1**

**
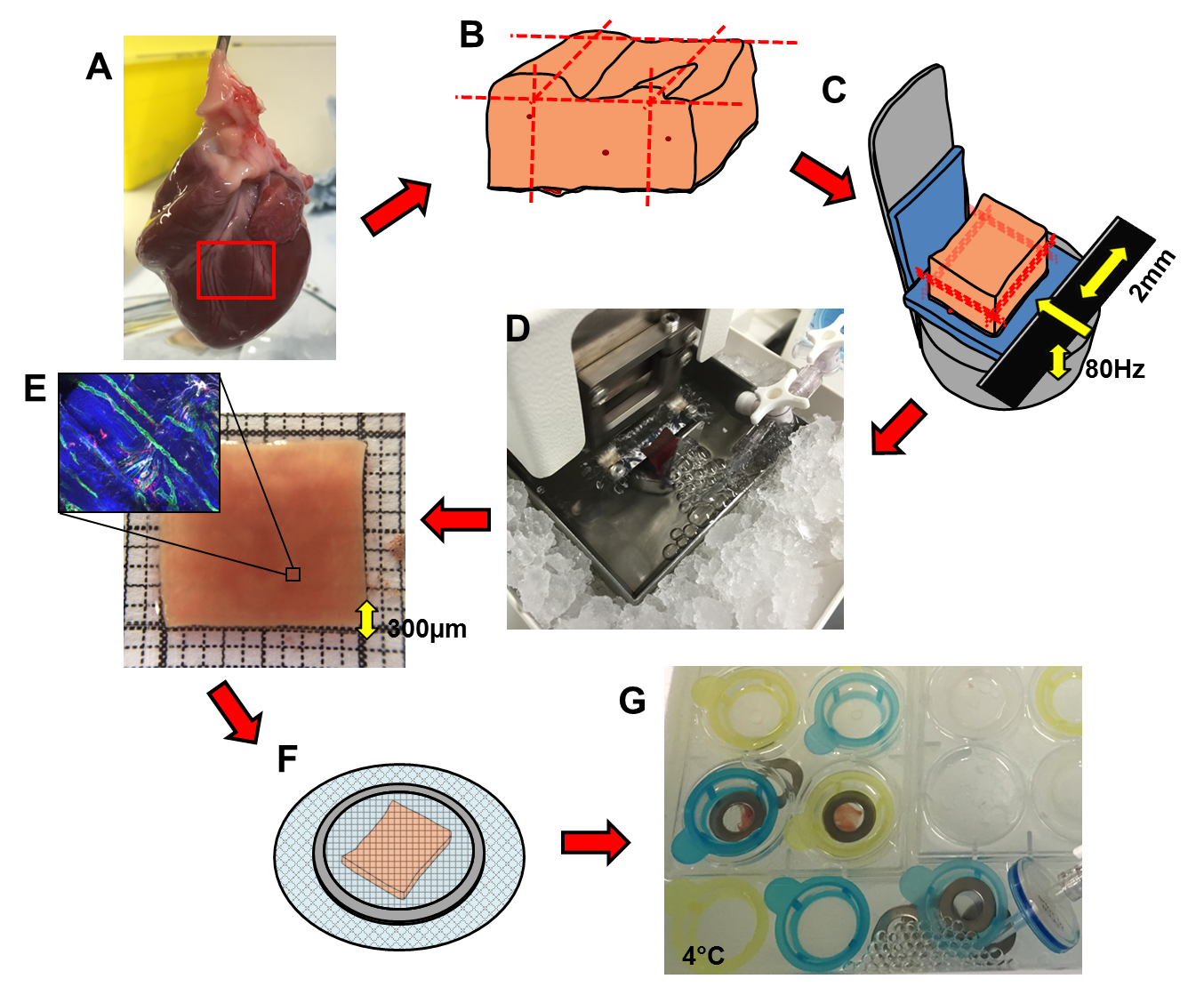
**

**Graphical representation of the myocardial slice preparation.** A) A tissue block is cut from the left ventricular free wall, B) the block is trimmed to obtain a 1cm^3^ cube of tissue C-E) which is sliced in 300µm sections using a vibrating ceramic blade. F,G) The myocardial slices are kept in a bath, under mesh holders, in 4°C oxygenated Tyrode Solution containing BDM.

**SUPP FIG**


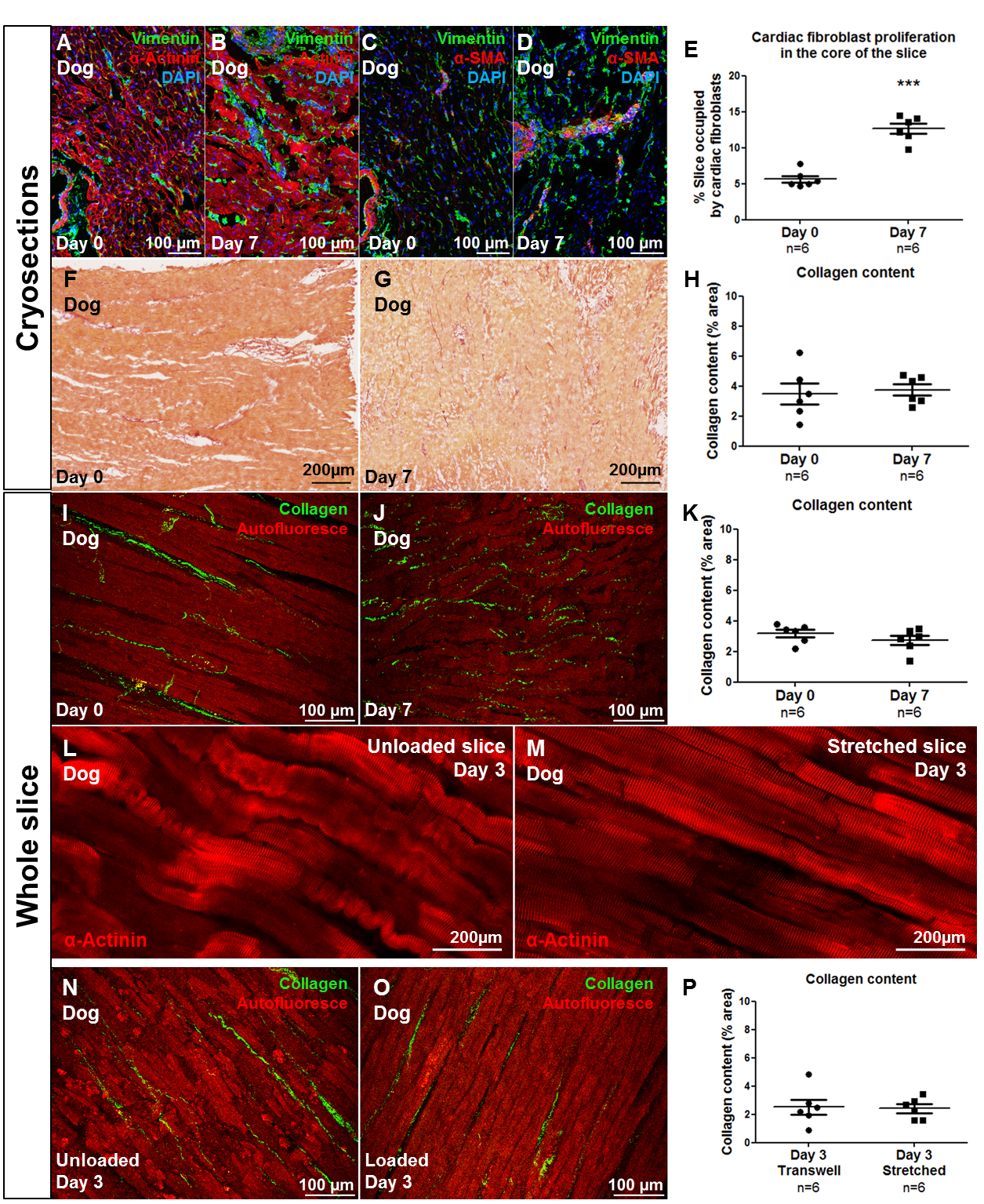


**Cardiac fibroblast proliferation and collagen content quantification of culture myocardial slices.** CF proliferation in the centre of the slice was studied using 15µm cryosection of myocardial slices. A,B) 15µm cryosection were labelled with α-Actinin and Vimentin to investigate CFs proliferation within the myocardium. C,D) The staining for Vimentin and αSMA was used to visualise the location of CFs near blood vessels. E) Cardiac fibroblasts proliferation in the centre of the slice was significantly higher in day 7 cultured slices compared to freshly prepared slices. F-H) PicroSirius Red staining and collagen content quantification of freshly prepared and culture (7 days) myocardial slices 15µm sections. I-K) Second harmonic generation imaging and collagen content quantification were used to study freshly prepared and *in vitro* cultured (7 days) whole myocardial slices. L,M) Representative images of tissue organization and sarcomeric structure of day 3 myocardial slices. N-P) Second harmonic generation imaging and collagen content quantification of day 3 myocardial slices cultured in loaded and unloaded condition. The application of mechanical load on myocardial slices preserved architecture in the sarcomeric structure and tissue organization but does not affect collagen content compared to unloaded slices. 6 slices prepared from at least 3 different experiments were used to quantify collagen content with hisological sections or SHG.4 images per slice were acquired.

**SUPP FIG 3**

**
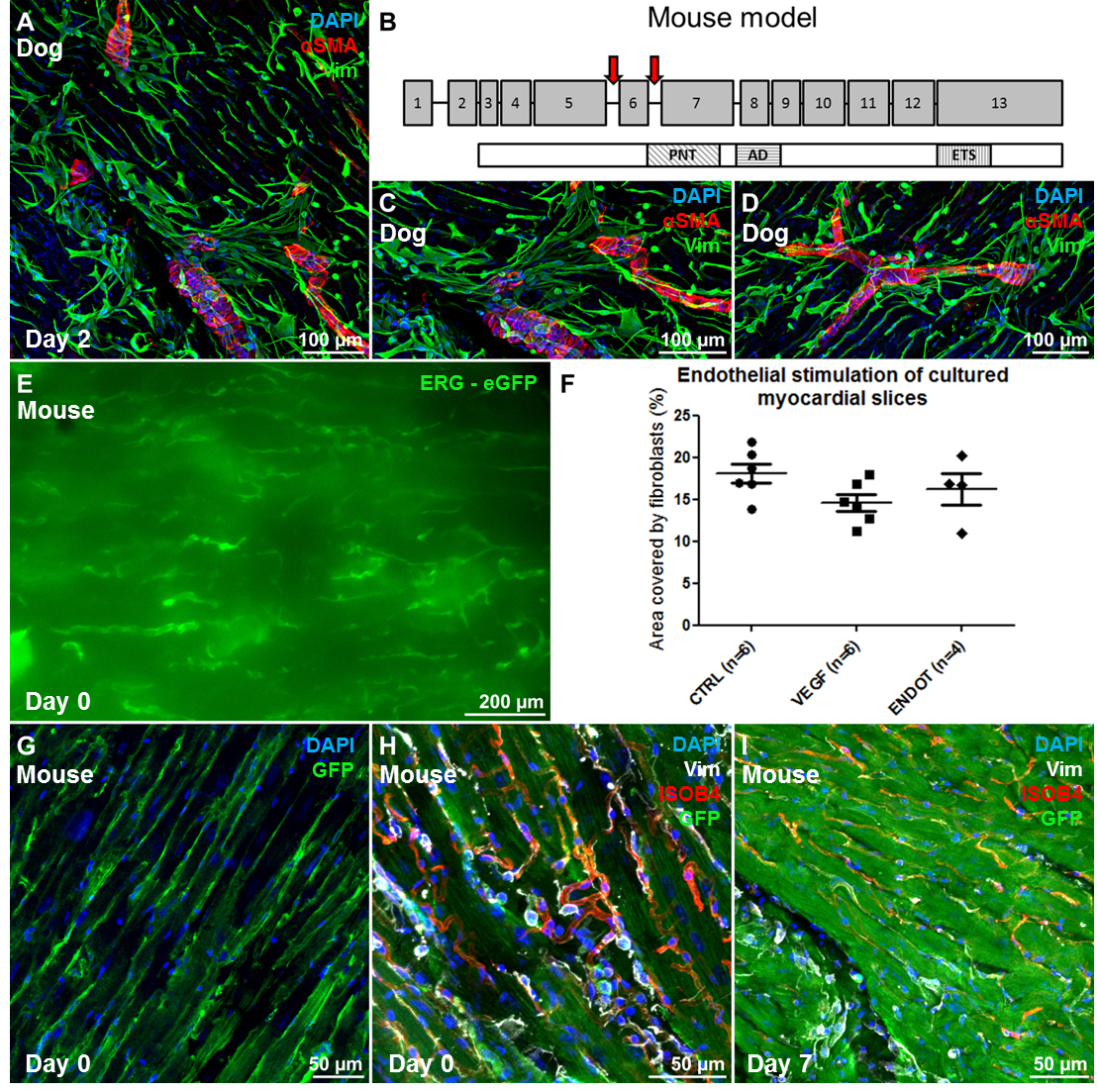
**

In canine and human slices we observed high densities of VIM+/αSMA- cells proliferating around blood vessels and migrating away from blood vessels; additionally, VIM+/αSMA- cells proliferate out of the interstitial space. (A, C, D). To investigate the role of EndMT we used Pdgfb-iCreER eGFP mice (B). Living mouse myocardial slices observed with a fluorescent microscope showed GFP+ vessels (E). The dog myocardial slices were treated with VEGF and endothelial medium to stimulate the endothelial cells proliferation but no differences in VIM+ proliferation were observed (F). After PFA fixation the mouse cardiac slices acquired to be stained with the GFP antibody (green) (G). Freshly prepared slices stained for VIM, ISOB4 and GFP showed co expression of GFP with Iso-B4 (red) confirming that endothelial cells were GFP+ (H), whereas fibroblasts (VIM+; white) cells were GFP-. (I) After 7 days the proliferating cells were VIM+/GFP- suggesting that EndMT is not involved in fibroblasts activation. Each experiment was repeated at least 3 times, 3 images were acquired from each slice and the data obtained were averaged before statistical analysis.

**SUPP FIG 4**


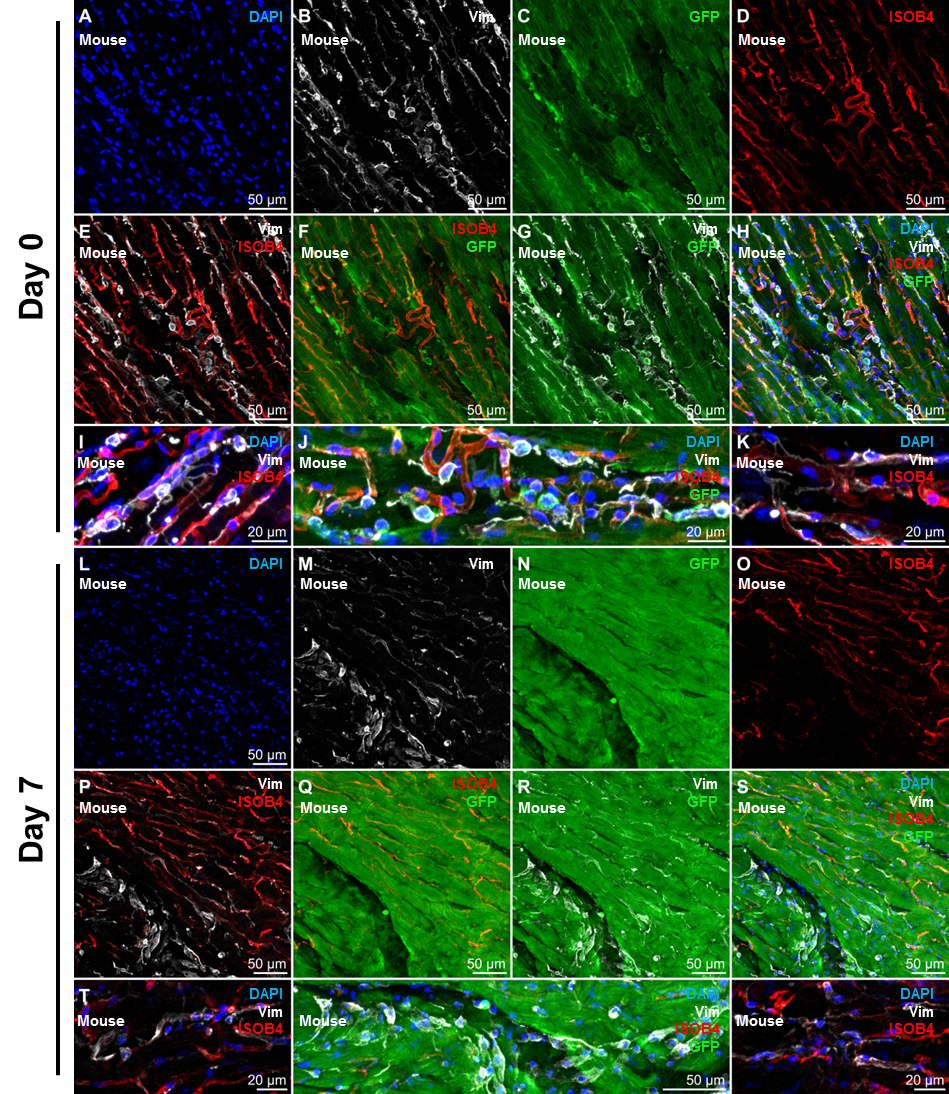


Histology and immunofluorescence staining of Day 1 (A-K) and Day 7 (L-V) mouse myocardial slices. The colours have been split and then merged to demonstrate co-localisation of antibodies. Figures I-K and T-V are representative areas at a higher magnification. VIM=white, GFP=green, ISOB4=red. Each experiment was repeated at least 3 times.

**SUPP FIG 5**

**
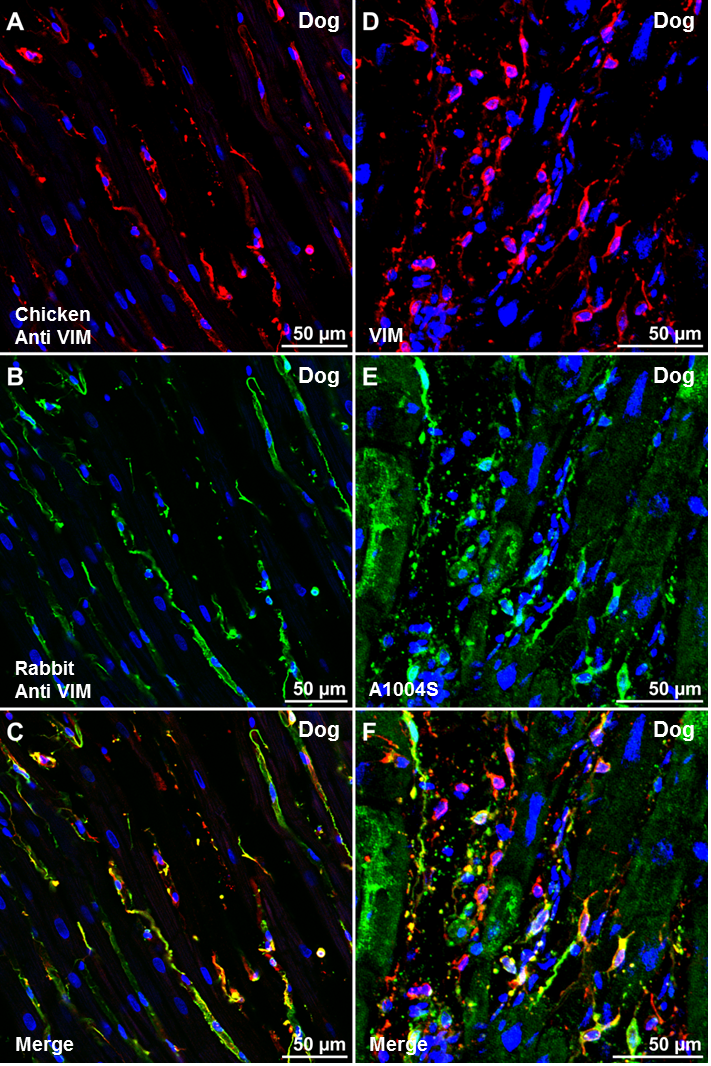
**

Immunofluorescence staining of canine myocardial slices for the fibroblast marker Vimentin and FSP1. A-C) Two VIM antibodies (chicken and rabbit) from two companies (Sigma and Thermo Scientific) were tested and the signal co-localised. (D-F) The antibody for S100A4 (Fibroblast-specific protein 1) was combined to VIM and most of the VIM+ cells also expressed S1004A. Each experiment was repeated at least 3 times.

**SUPP FIG 6**

**
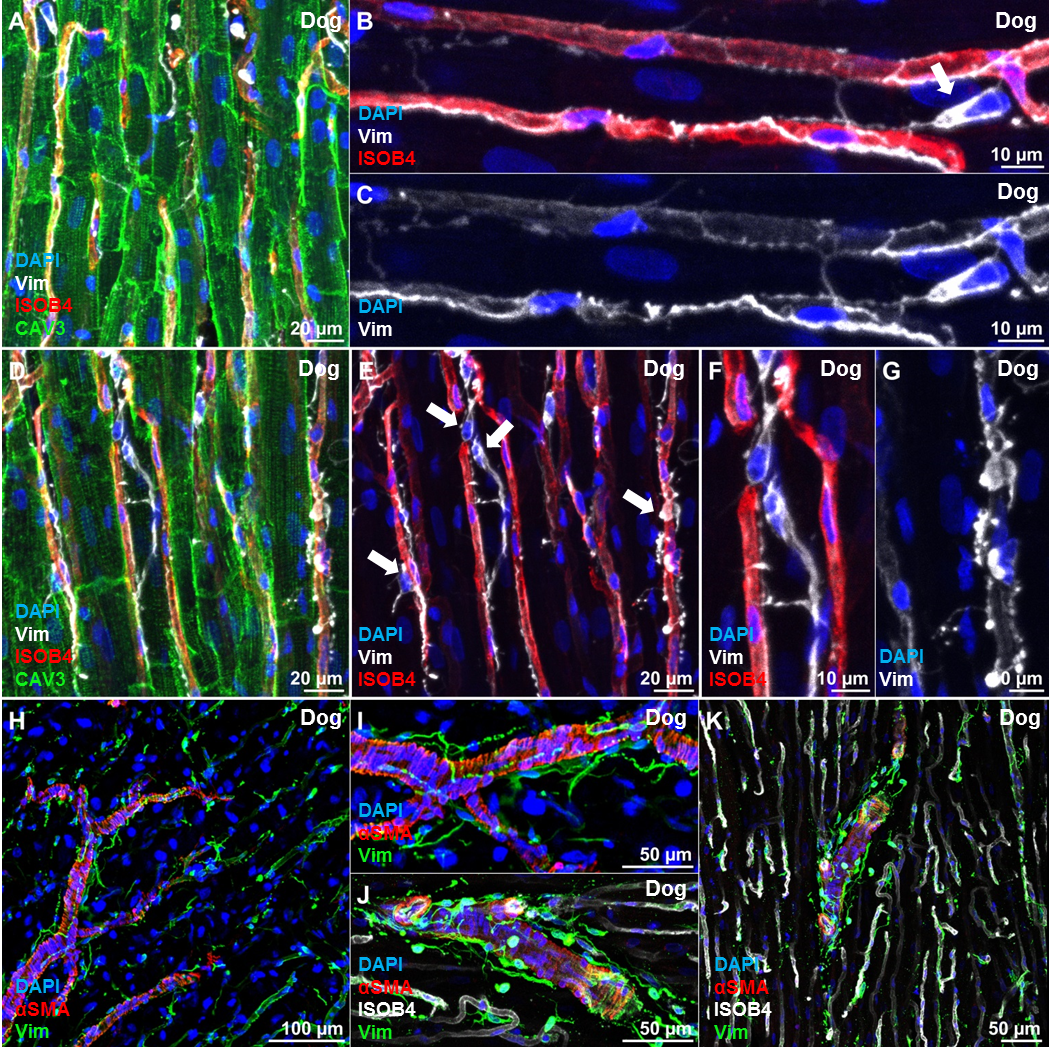
**

Immunofluorescence staining of freshly prepared dog myocardial slices (CAV3 green, VIM white, ISOB4 red). The fibroblasts have a thin and elongated cytoplasm (indicated with white arrows) with cytoplasmic protrusions that extend longitudinally and around the endothelium (A-G). Near larger vessels CFs (VIM+, green) are more abundant, they surround the smooth muscle cells (αSMA+, red) and there is no evidence of contact with the endothelium. Each experiment was repeated at least 3 times.
